# Functional Characterization of a 2OGD Involved in Abietane-Type Diterpenoids Biosynthetic Pathway in *Salvia miltiorrhiza*

**DOI:** 10.3389/fpls.2022.947674

**Published:** 2022-07-07

**Authors:** Zhimin Hu, Li Ren, Junling Bu, Xiuyu Liu, Qishuang Li, Wending Guo, Ying Ma, Jian Wang, Tong Chen, Ling Wang, Baolong Jin, Jinfu Tang, Guanghong Cui, Juan Guo, Luqi Huang

**Affiliations:** ^1^State Key Laboratory of Dao-di Herbs, National Resource Center for Chinese Materia Medica, China Academy of Chinese Medical Sciences, Beijing, China; ^2^School of Pharmaceutical Sciences, Henan University of Chinese Medicine, Zhengzhou, China

**Keywords:** *Salvia miltiorrhiza*, abietane-type diterpenoids, Fe(II)/2-oxoglutarate-dependent dioxygenase, hydroxylation, site-directed mutagenesis

## Abstract

*Salvia miltiorrhiza* is one of the most commonly used Chinese medicinal herbs. Tanshinones, the most abundant lipid-soluble bioactive constituents of *S. miltiorrhiza*, are a class of structural highly oxidized abietane-type diterpenoids with multiple pharmacological activities. Although several enzymes, including diterpene synthase, cytochrome P450, and Fe(II)/2-oxoglutarate-dependent dioxygenase (2OGD), have been functionally characterized in biosynthesis of abietane-type diterpenoids, the highly oxidized structure and complex secondary metabolic network of tanshinones imply that more oxidases should be characterized. Here, we identified a new 2OGD (Sm2OGD25) from *S. miltiorrhiza*. Molecular cloning and functional studies *in vitro* showed that Sm2OGD25 could catalyze the hydroxylation of sugiol at C-15 and C-16 positions to produce hypargenin B and crossogumerin C, respectively. The phylogenetic analysis of the DOXC family demonstrated that Sm2OGD25 belongs to the DOXC54 clade. Furthermore, structural modeling and site-directed mutagenesis characterization revealed the importance of the hydrogen-bonding residue Y339 and the hydrophobic residues (V122, F129, A144, A208, F303, and L344) in substrate binding and enzyme activity. This study will promote further studies on the catalytic characterization of plant 2OGDs and the secondary metabolic biosynthesis network of diterpenoids.

## Introduction

*Salvia miltiorrhiza* is one of the most commonly used Chinese herbal medicine. Its root and rhizome are used as medicine. It is clinically used in the treatment of coronary heart disease, angina pectoris, ischemic stroke, and other diseases, with remarkable curative effects, and extensively studied (Fang et al., 2018). Pharmacological studies show that *S. miltiorrhiza* has the effects of antibacterial, antitumor, sedative, analgesic, hepatoprotective, and others (Zhou et al., 2019). Tanshinone IIA, the most abundant lipid-soluble component in *S. miltiorrhiza*, and its water-soluble derivative sodium tanshinone IIA sulfonate (STS) were widely used in the clinic for anti-inflammatory activity, antioxidant activity, antifibrosis activity, and cardiovascular effects (Zhou et al., 2019; Bi et al., 2021).

**FIGURE 1:**
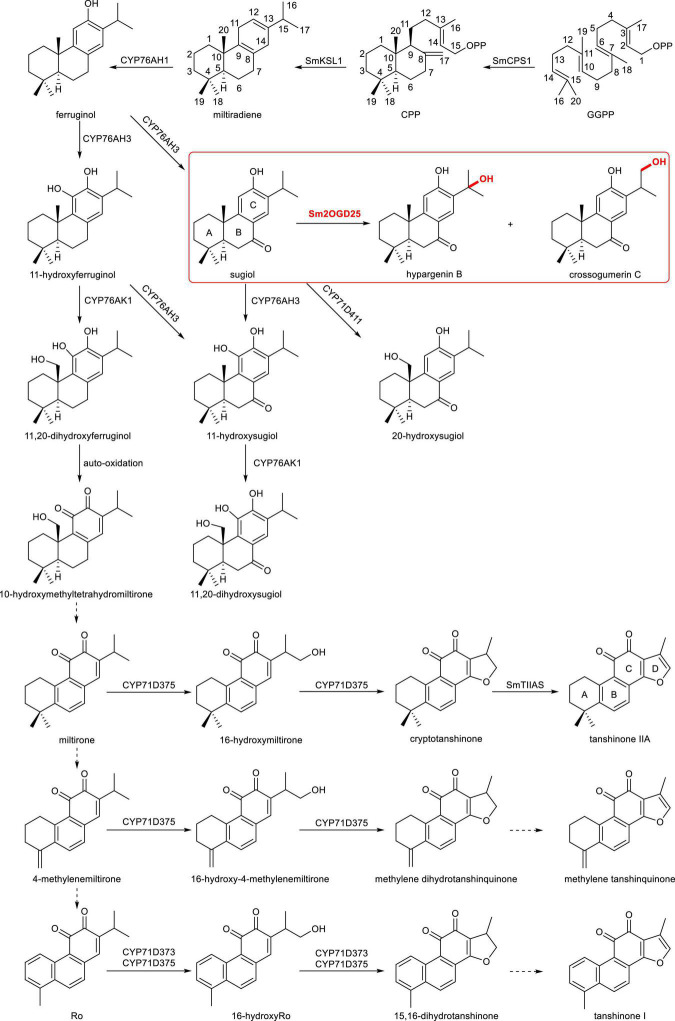
Proposed biopathways of tanshinone-related diterpenoid biosynthesis in *Salvia miltiorrhiza*. Solid arrows indicate the established relationships, and dashed arrows indicate hypothetical relationships.

Tanshinones are a class of structural highly oxidized abietane-type diterpenoids with various biological activities, including antibacterial, antioxidant, and anti-inflammatory activities, as well as cardiovascular and cerebrovascular protective effects (Wang et al., 2007; Zhang et al., 2012; Li et al., 2018), which inspired many studies toward their biosynthesis. Tanshinones biosynthesis pathway requires the skeleton formation, structural oxidation modification, aromatization, demethylation, and formation of furan ring (Figure 1) (Wu et al., 2012; Hu et al., 2021). The *S. miltiorrhiza* copalyl pyrophosphate synthase 1 (SmCPS1) and *S. miltiorrhiza* kaurene synthase-like 1 (SmKSL1) sequentially catalyze *(E, E, E)*-geranylgeranyl diphosphate (GGPP) to produce miltiradiene, the precursor of tanshinones (Gao et al., 2009). Subsequently, the cytochrome P450 (CYP) CYP76AH subfamily enzymes (CYP76AH1 and CYP76AH3) hydroxylate (C-12 and C-11 hydroxylation) and aromatize the C-ring, followed by C-20 hydroxylation by CYP76AK1 (Guo et al., 2013, 2015). It is noted that the crossover of substrates of CYP76AH3 and CYP76AK1 can lead to the bifurcation of the tanshinone biosynthetic pathway. Recently, three CYP71D enzymes have been characterized. CYP71D375 and CYP71D373 were found to catalyze hydroxylation at C-16 and 14,16-ether heterocyclization to form the dihydrofuran ring (D-ring), whereas CYP71D411 catalyzes C-20 hydroxylation (Ma et al., 2021). In addition, CYP76AK2 and CYP76AK3, two highly homologous enzymes to CYP76AK1, showed the same expression profile as *CYP76AK1*, indicating that these two enzymes might be involved in tanshinone biosynthesis (Li et al., 2021). The promiscuity of these identified CYPs suggests that tanshinones may be synthesized *via* a complicated metabolic network.

Apart from CYP450, the Fe(II)/2-oxoglutarate-dependent dioxygenase (2OGD) superfamily, as the second-largest enzyme family in plants, also catalyze a variety of reactions in natural product biosynthesis, including hydroxylation, halogenation, endoperoxidation, desaturation, and ring transformation (Kawai et al., 2014; Hagel and Facchini, 2018; Herr and Hausinger, 2018). Highly oxidized tanshinones indicate that not only CYP450s but also 2OGDs might be involved in the biosynthesis of tanshinones. Genome-wide, gene expression, and evolutionary analyses of the 2OGD superfamily in *S. miltiorrhiza* revealed that they facilitate the structural complexity of tanshinones (Xu and Song, 2017). Specifically, 2OGD5 was found to decrease the accumulation of miltirone, cryptotanshinone, and tanshinone IIA in RNAi transgenic lines, though the exact catalytic activity was missing. Recently, a 2OGD SmTIIAS (Sm2-ODD14) was identified to catalyze the dehydrogenation of dihydrofuran ring (D-ring), converting cryptotanshinone to produce the major active ingredient in *S. miltiorrhiza*, tanshinone IIA (Song et al., 2021) (Figure 1). This indicates that the 2OGDs also play roles in the biosynthetic network of abietane-type diterpenoids. Thus, more novel 2OGDs should be mined and characterized to understand the complex secondary metabolic network of tanshinones.

**FIGURE 2:**
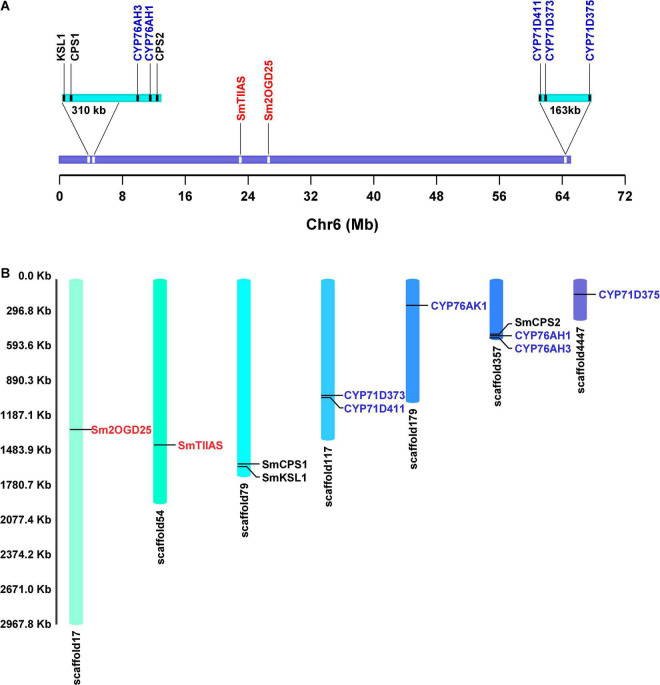
The positions of identified genes on pseudochromosome 6 and scaffolds. **(A)** Gene location of identified *diTPS*, *CYP450*, and *2OGD* genes on pseudochromosome 6. **(B)** The positions of 11 identified genes on scaffolds. Genes in black, blue, and red are functional *diTPS*, *CYP450*, and *2OGD* genes.

In previous studies, we discovered a large biosynthetic gene cluster in pseudochromosome 6 of *S. miltiorrhiza*, which includes all of the identified genes that participated in tanshinone biosynthesis except *CYP76AK1* (Ma et al., 2021) (Figure 2). In this study, we mainly mined and identified the *2OGD* genes of *S. miltiorrhiza* through genome analysis combined with the analysis of pseudochromosome 6. Functional studies *in vitro* identified a new Sm2OGD25 that could catalyze the hydroxylation of sugiol. The phylogenetic analysis of Sm2OGD25 with the DOXC family was analyzed. Furthermore, the key residues were also investigated by structural modeling, molecular docking, and site-directed mutagenesis. This study further facilitates the metabolic biosynthesis network of diterpenoids and provides a reference for the subsequent study of terpenoid-related 2OGD genes.

## Materials and Methods

### Plant Materials and General Remarks

The biennials *S. miltiorrhiza* at full-bloom stage used in this study were sampled from Henan Academy of Agricultural Sciences (Zhengzhou, Henan Province, China) and transported to the laboratory with soil in September, 2020. After washing, they were divided into roots, stems, leaves, and flowers, frozen with liquid nitrogen immediately after collection, and stored at –80°C. Sugiol (**1**) and other substrates (**2**–**25**) used in this study were purchased from Shanghai YuanYe Biotechnology Co., Ltd. (Shanghai, China) unless otherwise specified. Methanol and acetonitrile (Fisher Scientific, United States) were of high-performance liquid chromatography (HPLC) grade. All other chemicals and reagents were purchased from Sigma-Aldrich (St. Louis, MO, United States) and Beijing Chemical Corporation (Beijing, China) unless otherwise specified.

### Candidate Gene Screening and Cloning of *Sm2OGD* Genes From *S. miltiorrhiza*

*2OGD* genes with “oxoglutarate/ketoglutarate/non-haem” were screened from genome annotation data. Basic Local Alignment Search (Blast) comparison was performed on pseudochromosome 6 using the BioEdit software, and *2OGD* genes with e-value less than 1e^–20^ were screened. Candidate genes of *Sm2OGDs* with open reading frame (ORF) greater than 800 bp were cloned.

The total RNA of *S. miltiorrhiza* root was reverse-transcribed to cDNA with Invitrogen™ SuperScript™ III Reverse Transcriptase (18080, Thermo Fisher, United States). Using the cDNA library as a template, the full-length cDNA fragments of *Sm2OGDs* were amplified by PCR using 2 × TransStart^®^ KD Plus PCR SuperMix (AS301, Transgen Biotech, China) with the gene-specific primer pairs (Supplementary Table 1). The thermal cycling parameters were initially denaturized at 94°C for 3 min, followed by 35 cycles of 94°C for 30 s, 53°C for 30 s, and 68°C for 2 min, and a final extension at 68°C for 10 min.

### Heterologous Expression of *Sm2OGDs* in *Escherichia coli* and Protein Purification

The amplification products of *Sm2OGDs* were subcloned into expression vector pET-28a(+) vector (Invitrogen, United States) with pEASY^®^ -Basic Seamless Cloning and Assembly Kit (CU201, Transgen Biotech, China). After verification of the sequences (Ruibiotech, Beijing, China), the recombinant plasmid pET-28a(+)-Sm2OGDs was transformed into *E. coli* BL21(DE3) (TianGen Biotech, China) for heterologous expression. *E. coli* BL21(DE3) transformed with empty pET-28a(+) was employed as control. *E. coli* cells were cultured in 1 L Luria-Bertani medium containing 50 μg/ml kanamycin at 37°C with shaking (200 rpm) until OD_600_ reached 0.4–0.6, and then induced by 0.4 mM isopropyl-β-D-thiogalactopyranoside for protein expression. After 20 h of incubation at 16°C, the cell pellets were collected by centrifugation (8,000 rpm for 5 min at 4°C), and resuspended in 50 ml lysis buffer (pH 7.5, 50 mM Tris–HCl, 300 mM NaCl), and broken up at 2–6°C by a cryogenic homogenizer. After centrifugation (12,000 rpm for 30 min at 4°C), the cell debris was removed and the crude enzyme was obtained. The supernatant was subsequently mixed with 2 ml Ni-NTA resin (TransGen Biotech, China) and incubated for 1 h in an ice bath. Then, the mixture was applied to an Affinity Chromatographic Column (TransGen Biotech, China), which was pre-equilibrated with lysis buffer. The target recombinant protein was eluted by elution buffer containing 100 mM imidazole. The purified protein was concentrated and desalted by a 30 kDa ultrafiltration tube (Solarbio, China) with storage buffer (pH 7.5, 50 mM Tris–HCl, 300 mM NaCl, 20% glycerol), after sodium dodecyl sulfate polyacrylamide gel electrophoresis (SDS-PAGE) analysis.

### Functional Characterization of the Recombinant Sm2OGDs

The enzyme assay was performed in a reaction mixture (100 μl) comprising of 0.2 mM substrate, 1 mM 2OG, 1 mM L-ascorbic acid, 100 μM Fe(NH_4_)_2_●(SO_4_)_2_●6H_2_O, and 200 μg of the purified pET-28a(+)-Sm2OGDs in reaction buffer (50 mM Tris–HCl, 300 mM NaCl, pH 7.5). The reaction was incubated at 28°C for 12 h and stopped by adding 200 μl pre-cooled methanol and centrifuged at 12,000 rpm for 30 min for further ultra-performance liquid chromatography coupled with quadrupole time-of-flight tandem mass spectrometry (UPLC-QTOF-MS, Waters Technologies, Milford, MA, United States) analysis as described in Supplementary Table 2. *E. coli* BL21(DE3) transformed with empty pET-28a(+) was employed as control. To verify the dependence of Sm2OGD25 on 2OG and Fe^2+^, several enzymatic assays were performed as listed in Supplementary Table 4.

Effects of pH and temperature on the enzyme activity of Sm2OGD25 were tested by changing each of the activity assay conditions. All enzymatic reactions were individually performed in a final volume of 100 μl as described above. All experiments were performed in triplicate. The mixtures were analyzed by UPLC analysis as described in Supplementary Table 2.

### Ultra-Performance Liquid Chromatography Coupled With Quadrupole Time-of-Flight Tandem Mass Spectrometry Analysis

Analyses were analyzed by UPLC-QTOF-MS (Waters Technologies, Milford, MA, United States) for the identification of reactions. Samples were separated on an ACQUITY UPLC^®^ BEH C18 (2.1 mm × 100 mm, 1.7 μm, Waters Technologies, United States) protected with an ACQUITY UPLC^®^ BEH C18 guard column at 35°C, and the enzymatic products were eluted with a linear gradient condition given in Supplementary Table 2. Then, 5 μl of sample was injected into the system. The conversion rates in percent were calculated from peak areas of products and substrates as analyzed by UPLC.

Time-of-flight MS detection was performed with a Xevo G2-S QTOF (Waters) system equipped with electrospray ionization (ESI) operating in negative ion mode. Full-scan monitoring range was performed in the range of *m/z* 50–1,500. The other operating parameters were as follows: the scanning time of 0.1 s; the detection time of 18 min; and the cone voltage of 40 V; and the high-energy ramp collision voltage was 20–50 V. Data acquisition and processing were performed using the MassLynx version 4.1 software (Waters Corp., Milford, MA, United States).

### Preparative-Scale Reactions and Structural Identification of Hydroxylated Products

For isolation of the hydroxylated products of sugiol, 120 ml total enzyme reactions were performed in 50 mM reaction buffer (50 mM Tris-HCl, 300 mM NaCl, pH 7.5) containing 0.5 mM sugiol, 2 mM 2OG, 2 mM L-ascorbic acid, 0.2 mM Fe(NH_4_)_2_●(SO_4_)_2_6H_2_●O, and 250 mg of purified Sm2OGD25. The reactions were incubated for 12 h at 37°C and extracted with 240 ml ethyl acetate (EtOAc) for five times. The organic phase was collected, concentrated, and dissolved in 2 ml methanol. The hydroxylated products were subsequently separated by reversed-phase semi-preparative HPLC on Shimadzu LC-20AR preparative liquid chromatography system, with a YMC-Pack ODS-A column (250 mm × 10 mm I.D., S-5 μm, 12 nm). The enzymatic product **1a** was eluted with 60% acetonitrile (solvent B) in H_2_O (solvent A), while 45% acetonitrile (solvent B) in H_2_O (solvent A) was used for further purification of **1b**, with a flow rate of 2 ml/min in each case. For chemical structure characterization, NMR spectra were recorded on a Bruker Ascend™ 600 MHz spectrometer with CDCl_3_ as the solvent, using TMS as reference. Chemical shifts (δ) are given in parts per million (ppm) and coupling constants (*J*) are given in Hertz (Hz). ^1^H and ^13^C NMR data of hydroxylated products are shown in Supplementary Table 3.

### Phylogenetic Analysis

A phylogenetic tree was constructed using the neighbor-joining method with 1,000 bootstrap replicates. Construction of the tree was performed using the MEGA7.0 software^1^ using sequences aligned with ClustalW. All GenBank accession numbers used in this study are given in Supplementary Table 5.

### Structure Prediction and Molecular Docking

The structure model of Sm2OGD25 was predicted by AlphaFold2 with the default parameters (Laskowski et al., 1993; Senior et al., 2020; Jumper et al., 2021). Molecular docking was performed using the AutoDockTools-1.5.7 (ADT) software. The iron position was extracted from the reported *Arabidopsis* AtJOX2 complex (Zhang et al., 2021) (PDB ID: 6LSV) and optimized based on the coordinated conserved active sites (H229, D231, and H287 in Sm2OGD25). The structural formulas of 2OG and sugiol were saved as 3D structural formulas after energy optimization by the Chem3D software. The protein structure was hydrogenated and charged and saved in pdbqt format. The charge of the small-molecule ligand was checked and the flexible torsion bond was set by ADT. The protein activity center was predicted by the DeepSite^2^ online server, the docking box Grid box was set according to its prediction results, and other docking parameters keep the default values. All structure figures were plotted using the PyMOL 2.5 software.

### Site-Directed Mutagenesis and Functional Characterization of Mutants

Site-directed mutagenesis was constructed using the Fast Mutagenesis System Kit (FM111, Transgen Biotech, China) according to the instructions of the manufacturer. The primers are listed in Supplementary Table 1. Protein expression, purification, and functional characterization of all mutants were performed as described above for the wild-type (WT) Sm2OGD25.

## Results

### Mining of *2OGDs* From *Salvia miltiorrhiza*

In our previous study, the line bh2-7 that has been bred close to homozygosity was sequenced to obtain a high-quality genome sequence of *S. miltiorrhiza*. To identify the putative *2OGD* genes, the genome data were reanalyzed. A total of 122 *2OGD* genes annotated with “oxoglutarate/ketoglutarate/non-haem” were screened from the genome. All of the identified *CYP* genes involved in tanshinone biosynthesis except *CYP76AK1* were located on one pseudochromosome (∼65 Mb) defined as pseudochromosome 6 (Ma et al., 2021). Alignment analysis revealed that SmTIIAS, a 2OGD actor as dehydrogenase catalyzing the formation of tanshinone IIA, was also found to be localized on the pseudochromosome 6 (Figure 2). Therefore, we mainly focused on those *2OGD* genes located on pseudochromosome 6 to find out their roles in metabolic biosynthesis network of *S. miltiorrhiza*. A total of 26 *2OGD* genes with e-value less than 1e^–20^ with full-length ORF were screened out. Here, they were annotated as *Sm2OGD1–26*. Among them, 13 *Sm2OGDs* were cloned successfully using specific primers (Supplementary Table 1). These candidate *2OGDs* were used for heterologous expression for *in vitro* enzyme assay.

### Functional Characterization of Sm2OGDs

**FIGURE 3:**
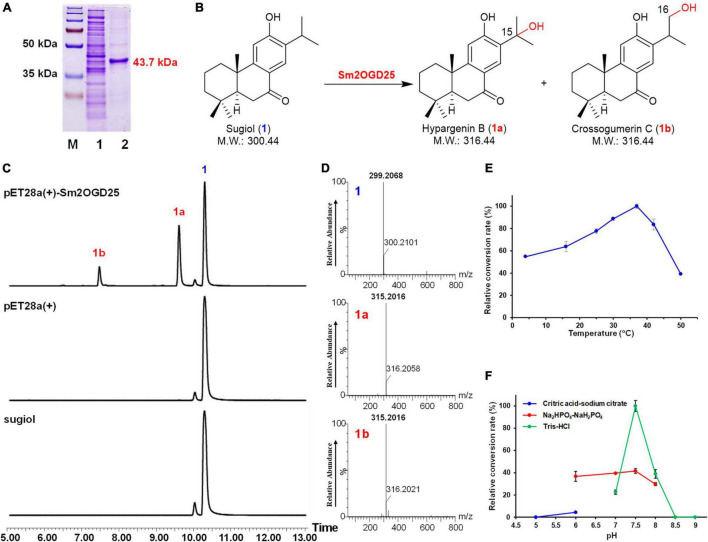
Functional characterization of recombinant Sm2OGD25. **(A)** SDS-PAGE of the His-tagged Sm2OGD25 purified by Ni-NTA affinity chromatography. M: protein marker; 1: crude protein of Sm2OGD25; 2: recombinant Sm2OGD25 (purity > 95%) fused with His-tag (predicted M.W.: 43.7 kDa). **(B)** Enzymatic reaction catalyzed by Sm2OGD25. **(C)** UPLC-QTOF-MS analysis of 1 and enzymatic product 1a and 1b. **(D)** (-)-ESI-MS spectra of 1, 1a, and 1b in the negative ion mode. The analysis conditions are described in Supplementary Table 2. Effects of temperature **(E)** and reaction buffer **(F)** on enzyme activity of Sm2OGD25.

To investigate the enzyme activities of Sm2OGDs, the *Sm2OGDs* were expressed in *E. coli* BL21(DE3). The purified proteins were verified by SDS-PAGE after Ni-affinity purification (Figure 3A). Functional studies *in vitro* were carried out with 2OG, Fe^2+^, and L-ascorbic acid as cofactors. A total of 25 abietane-type diterpenoids (**1**–**25**), which have been reported in *S. miltiorrhiza* and could be obtained, were employed as substrates (Supplementary Figure 1). The enzymatic mixtures were analyzed by UPLC-QTOF-MS. The enzyme extracted from *E. coli* BL21(DE3) transformed with pET28a(+) empty vector was used as the negative control. As a result, only Sm2OGD25 exhibited hydroxylation activity toward sugiol (**1**), which has been regarded as an intermediate involved in biosynthesis of tanshinones (Figures 3B–D). UPLC-QTOF-MS analysis of the enzymatic reaction products revealed two new products appeared at *t*_*R*_ 9.6 and 7.4 min. MS analysis showed that the pseudo-ion peak of **1a** and **1b** at *m/z* 315.2016 ([M-H]^–^) was 16 amu greater than that of sugiol, suggesting **1a** and **1b** were monohydroxylated products. To confirm the hydroxylation site, the products **1a** and **1b** were then prepared from a scaled-up enzymatic reaction. The enzyme products **1a** and **1b** were eluted with 60% acetonitrile and 45% acetonitrile, respectively. The prepared hydroxylation products were analyzed by ^1^H and ^13^C NMR (Supplementary Figures 2, 3 and Supplementary Table 3) to identify as hypargenin B (**1a**, 15-hydroxy sugiol) (Ulubelen et al., 1988; Yao et al., 2008; Wu et al., 2012) and crossogumerin C (**1b**, 16-hydroxy sugiol) (Miron-Lopez et al., 2014; Chen et al., 2019; Vo et al., 2020). This result demonstrated that Sm2OGD25 is a new enzyme that catalyzed the hydroxylation of sugiol (**1**) at C-15 and C-16 positions. This will provide a reference for the decoration of abietane-type diterpenoids.

### Effects of Cofactors, pH, and Temperature on the Enzyme Activity of Sm2OGD25

To verify the dependence of Sm2OGD25 on 2OG and Fe^2+^, several enzymatic assays were performed as listed in Supplementary Table 4. The results showed that no hydroxylated product was detected in the reaction without 2OG (reaction 4). When Fe^2+^ was absent from the reaction system, **1b** could not be detected and the conversion rate decreased because of trace amounts of Fe^2+^ from the purified protein (reaction 6). When ethylene diamine tetraacetic acid (EDTA) was added to the reaction system, no product was detected in the reaction with EDTA (reaction 7), or with Fe^2+^ and EDTA (reaction 8). These indicated that Sm2OGD25 is an Fe^2+^ and 2OG-dependent dioxygenase (Supplementary Figures 6, 7). Additionally, biochemical properties of Sm2OGD25 were investigated using **1** as the substrate (Figures 3E,F). Sm2OGD25 activity was tested in a temperature range of 4–50°C, and the optimal activity was detected at 37°C (Figure 3E). Analysis of the enzyme activity between pH 5.0 and 9.0 showed that the optimal pH value was observed at pH 7.5 (50 mM Tris–HCl, Figure 3F).

### Phylogenetic Analysis

**FIGURE 4:**
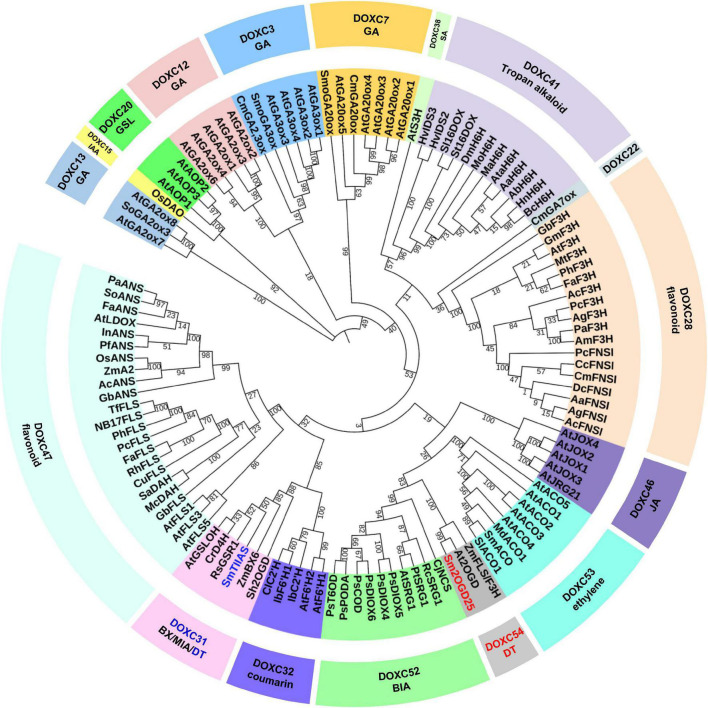
Phylogenetic tree analysis of Sm2OGD25 and other DOXCs. The accession numbers of sequences used in this study are shown in Supplementary Table 5. GA, gibberellin; IAA, indole-3-acetic acid; GSL, glucosinolate; SA, salicylic acid; JA, jasmonic acid; DT, diterpenoid; BIA, benzylisoquinoline alkaloid; BX, benzoxazinoid; MIA, monoterpenoid indole alkaloid.

It was revealed that most of the CYP450s involved in biosynthesis of labdane-related diterpenoids were clustered by phylogenetic analysis (Bathe and Tissier, 2019). This led the way into Lamiaceae labdane-related diterpenoid biosynthesis (Wang and Peters, 2022). Sm2OGD25 was the second functional characterized 2OGD in *S. miltiorrhiza.* It shows a relatively low-sequence identity of 24% with SmTIIAS. Low identity might result in different catalytic activity by using similar carbon skeleton substrates. 2OGDs of the DOXC class in six plants ranging from green algae to angiosperms were classified into 57 phylogenetic clades (DOXC1–57) (Kawai et al., 2014). To explore the evolutionary relationship of 2OGDs with the other plant 2OGDs that have been identified to be involved in the metabolism of gibberellins, phenylpropanoids, and alkaloids, a neighbor-joining phylogenetic tree was constructed. Sm2OGD25 was grouped into the DOXC54 clade with At2OGD and ZmFLS/F3H from *Arabidopsis thaliana* and *Zea mays*, respectively (Figure 4). It shares 70 and 53% sequence identity with At2OGD and ZmFLS/F3H, seperately (Supplementary Figure 8). Phylogenetic analysis revealed that Sm2OGD25 was clustered in the DOXC54 clade, as the first functionally characterized 2OGD in this clade. Furthermore, an assay of Sm2OGD25 orthologous proteins from other tanshinone-producing *Salvia* species showed that *S. scapiformis* and *S. bowleyana* have the same gene as *Sm2OGD25*, demonstrating that *Sm2OGD25* was conserved in *Salvia* species (Supplementary Figure 8).

### Structure Prediction and Substrate Binding Site of Sm2OGD25

**FIGURE 5:**
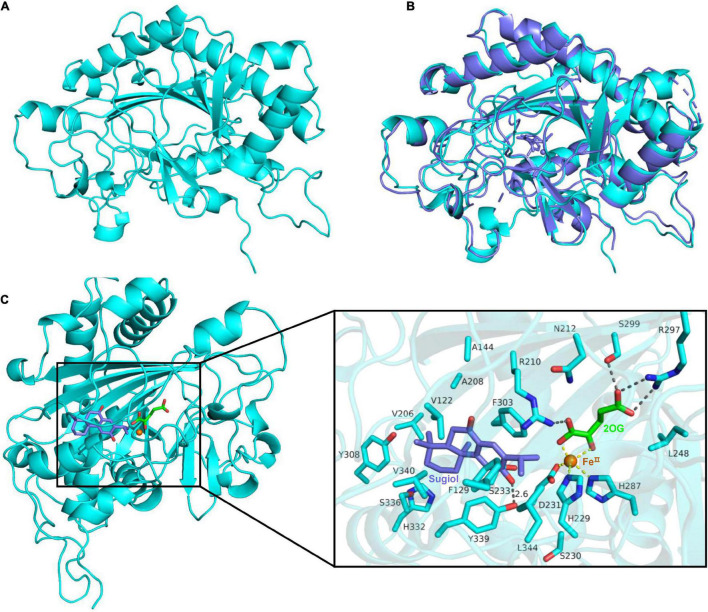
Predicted structure, structural comparison, and substrate-binding site of Sm2OGD25. **(A)** 3D structure of Sm2OGD25 predicted by AlphaFold2. **(B)** Structural alignment of Sm2OGD25 (colored in cyan) and AtJOX2 (PDB ID: 6LSV, colored in slate). The root mean square deviation (RMSD) value is 0.99 Å for 271 Cα atoms. **(C)** Structure modeling of Sm2OGD25. Sugiol (1) was docked into the potential binding pocket of Sm2OGD25. Residues that were close (< 5 Å) to sugiol (1) are displayed. All protein elements are shown as cartoons or sticks in cyan. Ligands and iron are shown in different colors. The key hydrogen bond interactions are labeled in black dashed lines. The coordination with iron is labeled in yellow dashed lines.

To probe the site related to the function of Sm2OGD25, function–structure relationship was analyzed by structural modeling, site-directed mutation, and enzyme assay (Figure 5). To investigate how substrate sugiol (**1**) binds to the active pocket of Sm2OGD25 for catalysis, the 3D structure of Sm2OGD25 was predicted by AlphaFold2 (Figures 5A,B) (Laskowski et al., 1993; Senior et al., 2020; Jumper et al., 2021). Sugiol was subsequently docked into the model by AutoDock (Figure 5C). The simulated Sm2OGD25 structure is overall very similar to that of AtJOX2 (Zhang et al., 2021) complex structure (PDB ID: 6LSV) with a root mean square deviation (RMSD) of 0.99 Å for 271 Cα atoms (Figure 5B). It adopts the double-stranded β-helix (DSBH) core and contains the conserved 2-His-1-Asp facial triad typically observed in members of 2OGDs (Hagel and Facchini, 2018; Islam et al., 2018). The iron atom is coordinated by the conserved residues H229, D231, and H287, while 2OG is bound to the side chains of R210, R297, and S299 through hydrogen bonds in Sm2OGD25. The docked results showed that substrate sugiol (**1**) is located on the opposite side of the conserved facial triad and the distances between the iron and the reaction site C-15 and C-16 of sugiol (**1**) are 4.1 and 4.7 Å (Figure 5C), respectively, which are close sufficient for hydrogen atom abstraction by C–H hydroxylation. This demonstrates that the model is in accordance with the experimental result that both C-15 and C-16 sites were hydroxylated.

### Site-Directed Mutagenesis of the Critical Residues

**FIGURE 6:**
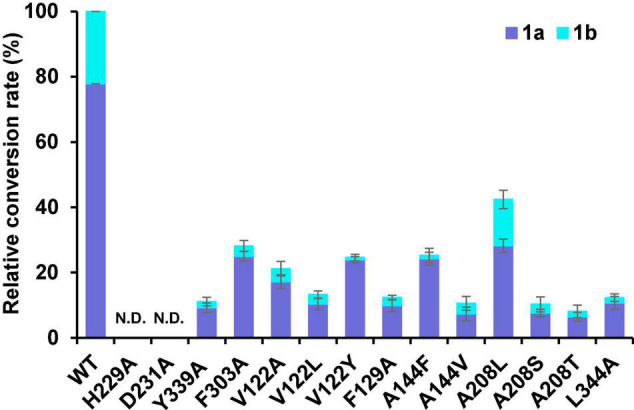
The relative conversion rates of wild-type (WT) Sm2OGD25 and mutants. *In vitro* assays of Sm2OGD25 and its variants using sugiol (1) as the substrate. N.D. represents mutants with no detected activity. Error bars indicate standard deviation of three independent replicates.

To further explore the potential active site residues of Sm2OGD25, site-directed mutagenesis was performed combined with the sequence alignment analysis (Figure 5 and Supplementary Figure 8). Analysis of the interaction between Sm2OGD25 and sugiol (**1**) revealed that the substrate is located in a relatively narrow active pocket, in which residue Y339 forms a hydrogen bond with the 12-OH of sugiol with a distance of about 2.6 Å, while residues V122, F129, A144, A208, F303, and L344 may play hydrophobic roles. Particularly, the benzene ring of residue F303 may stabilize the C-ring (benzene ring) of sugiol by a parallel mode similar to π–π stacking interaction. Together, these interactions stabilize the substrate binding conformation and orientation and facilitate the catalysis of Sm2OGD25. The hydroxylation activity of each mutant was measured using sugiol (**1**) as the substrate (Figure 6). Indeed, substitutions of H229 and D231 that form the coordination bonds with iron to A completely abolished enzyme activity. The Y339A mutant drastically decreased the hydroxylation activity, indicating that the hydrogen-bonding interaction mediated by Y339 plays an important role in substrate binding. The retained enzyme activity catalyzed by the F303A variant suggests that the aromatic ring of residue F303 is not crucial for Sm2OGD25 catalysis. Additionally, substitutions of the active site hydrophobic residues, V122, F129, A144, A208, and L344, also resulted in reduced enzyme activity. These results further support that the hydrogen-bonding and hydrophobic interactions mediated by the key residues are significant for substrate binding and Sm2OGD25 enzyme activity. Regrettably, we did not obtain mutations that accelerate the activity.

## Discussion

Oxidation is an important process to accelerate the biological activity of diterpenoids, such as tanshinones (Zhou et al., 2019; Bi et al., 2021). In decoration of diterpene skeletons, CYP450s have been proved to play essential roles. In our previous studies, six CYPs (CYP76AH1, CYP76AH3, CYP76AK1, CYP71D411, CYP71D373, and CYP71D375) have been identified to account for structural oxidative modification, including hydroxylation and carbonylation at different sites, and 14,16-ether heterocyclization (Gao et al., 2009; Guo et al., 2013, 2015; Ma et al., 2021). This also leads the way to Lamiaceae labdane diterpenoids biosyntheses such as forskolin (Pateraki et al., 2017) and carnosic acid (Ignea et al., 2016). In addition to CYP450s, 2OGD superfamily was also an important structural modification enzyme (Herr and Hausinger, 2018). Recently, SmTIIAS, a newly identified 2OGD, was found to dehydrogenate the dihydrofuran ring to form the furan ring of Tanshinone IIA (Song et al., 2021). There are 122 2OGDs in the genome of *S. miltiorrhiza*. The function of these 2OGDs in the secondary metabolite biopathway of *S. miltiorrhiza* was worth investigating. It was found that all of the identified genes except *CYP76AK1* that participated in tanshinone biosynthetic pathway are located on the pseudochromosome 6 of *S. miltiorrhiza* (Ma et al., 2021). In this study, we aimed to mine the *2OGD* genes located on the pseudochromosome 6 of *S. miltiorrhiza*. As a result, we identified a new 2OGD Sm2OGD25 that catalyzes the hydroxylation of sugiol (**1**) at C-15 and C-16 positions to produce hypargenin B (**1a**) and crossogumerin C (**1b**), respectively. The identification of Sm2OGD25 gene further confirms the significance of pseudochromosome 6 in tanshinone-related diterpenoid biosynthesis. It should be mentioned that CYP81AM1 from *Tripterygium wilfordii* could catalyze C-15 hydroxylation of dehydroabietic acid, an abietane-type diterpenoids in *T. wilfordii* (Wang et al., 2021). However, we could not find a homologous in *S. miltiorrhiza*. Functional redundancy of CYP450s and 2OGDs evolved from different species resulting in the complexity of terpenoids still needs extensive investigation.

2OGD superfamily is the second-largest enzyme family in the plant genome ranking only second to cytochrome P450 monooxygenase, whose members are involved in various oxidation reactions (Islam et al., 2018). 2OGDs were phylogenetically divided into DOXA, DOXB, and DOXC classes according to the similarity of amino acid sequence (Kawai et al., 2014). DOXA is involved in oxidative demethylation of alkylated nucleic acids and histones. The DOXB class is mainly involved in proline hydroxylation in protein post-translation modification. Most of the 2OGDs in terrestrial plants were classified into DOXC class, which includes those 2OGDs involved in biosynthesis and catabolism of several important phytohormones, as well as the diversified secondary metabolites.

Compared to DOXA and DOXB, 2OGDs in DOXC seems considerable variable. It could be divided into 57 phylogenetic clades. Expansion of DOXC is supposed to be related to secondary metabolisms of land plants (Kawai et al., 2014). In this study, we aligned Sm2OGD25 with 2OGDs in DOXC class. Phylogenetic analysis indicated that the function of 2OGDs in DOXC was phylogenetically clustered (Kawai et al., 2014). This includes 2OGDs clades involved in gibberellin biosynthesis that are conserved in vascular plants (Araújo et al., 2014) and ethylene biosynthesis that were shared in seed plants (Tohge et al., 2013). Several clades of DOXC class in angiosperms were specific to secondary metabolism, indicating that they contribute to the diversity and complexity of secondary metabolites in terrestrial plants. Most of the functionally characterized 2OGDs in the phylogenetic tree were involved in biosynthesis of flavonoids (DOXC28 and DOXC47). DOXC31, DOXC41, and DOXC52 were reported to be involved in biosynthesis of alkaloids (Araújo et al., 2014). However, 2OGDs involved in the biosynthesis of terpenoids, the most abundant component in plants, have been rarely studied especially in secondary metabolite biosynthesis. Sm2OGD25 and SmTIIAS were clustered separately in the phylogenetic tree as there were no more than 24% amino acid sequence identities. The catalytic activity of these two 2OGDs enriches the role of 2OGDs in the diversity of terpenoids in plants.

A total of 132 2OGDs were identified in *S. miltiorrhiza* by a genome-wide strategy. RNAi of 2OGD5 was found to cause the decrease of miltirone, cryptotanshinone, and tanshinone IIA by metabolomic analysis, but the crucial catalytic function was missing (Xu and Song, 2017). SmTIIAS and its orthologous 2OGDs from *Salvia* species were identified to catalyze the desaturation of the dihydrofuran ring in cryptotanshinone and isocryptotanshinone. SmTIIAS was clustered into the DOXC31 (also namely D4H/BX6) clade involved in the hydroxylation of DIBOA, glucosinolates, and monoterpenoid indole alkaloid with 50% sequence identities. D4H/BX6 clade is the largest DOXC group in *S. miltiorrhiza* and is supposed to participate in the tanshinone biosynthesis based on a genome-wide strategy (Xu and Song, 2017). Limited by the rare reports of 2OGDs in terpenoid biosynthesis pathway, the function of 2OGDs in D4H/BX6 clade in biosynthesis of tanshinones still needs further investigation. Phylogenetic analysis revealed that Sm2OGD25 was clustered in the DOXC54 clade as the first functionally characterized 2OGD in this clade. To our knowledge, Sm2OGD25 is the first functionally characterized 2OGD in this clade, which will guide the further mining and identification of other DOXC54 members. SmTIIAS responsible for dehydrogenation of cryptotanshinone to tanshinone TIIA was grouped into the DOXC31 clade (Figure 4), demonstrating that 2OGDs from different DOXC clades may participate in the abietane-type diterpenoids metabolic network, laying a foundation for the study of DOXC-class 2OGDs in diterpenoid biosynthesis. Phylogenetic analysis of functionally characterized 2OGDs would provide direction for gene functional characterization of 2OGDs.

In summary, a new 2OGD (Sm2OGD25) was identified from *S. miltiorrhiza*. Sm2OGD25 could catalyze the hydroxylation of sugiol at C-15 and C-16 positions to produce hypargenin B and crossogumerin C, respectively, which may play an important role in diterpenoids metabolic biosynthetic network. Structure simulation by AlphaFold2, molecular docking, and site-directed mutagenesis characterization revealed the importance of the hydrogen-bonding residue Y339 and the hydrophobic residues (V122, F129, A144, A208, F303, and L344) in substrate binding and enzyme activity. This enhanced our understanding of structure–function and structure–reactivity relationship of these important decoration enzymes. This study will facilitate further studies on the catalytic characterization of plant 2OGDs and contribute to enriching the understanding of the abietane-type diterpenoids biosynthesis network. Further studies will be conducted to mine more biosynthetic genes from pseudochromosome 6 to provide insights into the complex metabolic network of tanshinones.

## Data Availability Statement

The datasets presented in this study can be found in online repositories. The data presented in the study are deposited in the Figshare repository, doi: 10.6084/m9.figshare.20099012.

## Author Contributions

ZH, JG, and LH conceived and designed the experiments. ZH and LR performed the experiments. JB and XL contributed to the data analysis. QL and WG contributed to the sample collection. YM, JW, TC, and LW helped in data analysis. ZH and JG wrote the manuscript. All authors discussed the results, commented on the manuscript, read, and approved the final manuscript.

## Conflict of Interest

The authors declare that the research was conducted in the absence of any commercial or financial relationships that could be construed as a potential conflict of interest.

## Publisher’s Note

All claims expressed in this article are solely those of the authors and do not necessarily represent those of their affiliated organizations, or those of the publisher, the editors and the reviewers. Any product that may be evaluated in this article, or claim that may be made by its manufacturer, is not guaranteed or endorsed by the publisher.

## Footnotes

1
http://www.megasoftware.net/

2
https://playmolecule.com/deepsite/
