# Supplementary material for: Functional Characterization of a 2OGD Involved in Abietane-Type Diterpenoids Biosynthetic Pathway in Salvia miltiorrhiza
Source: Front Plant Sci. 2022 Jul 7;13:947674. doi: 10.3389/fpls.2022.947674 (PMC9301305; doi:10.3389/fpls.2022.947674)
Supplement: Supplementary file 1 [file Data_Sheet_1.docx]

Supplementary Material

**Supplemental Information**

**Functional characterization of a 2OGD involved in abietane-type diterpenoids biosynthetic pathway in *Salvia miltiorrhiza***

**Zhimin Hu**^1^**, Li Ren**^1^**, Junling Bu**^1^**, Xiuyu Liu**^1,2^**, Qishuang Li**^1^**, Wending Guo**^1^**, Ying Ma**^1^**, Jian Wang**^1^**, Tong Chen**^1^**, Ling Wang**^1^**, Baolong Jin**^1^**, Jingfu Tang**^1^**, Guanghong Cui**^1^**, Juan Guo**^1^**^*^ and Luqi Huang**^1^**^*^**

^1^State Key Laboratory of Dao-di Herbs, National Resource Center for Chinese Materia Medica, China Academy of Chinese Medical Sciences, NO.16 Neinanxiaojie, Dongzhimen, Beijing, China

^2^School of Pharmaceutical Sciences, Henan University of Chinese Medicine, Zhengzhou, Henan Province, China

*Correspondence to: J. Guo, and L. Huang, State Key Laboratory of Dao‐di Herbs, National Resource Center for Chinese Materia Medica, China Academy of Chinese Medical Sciences, No 16 Neinanxiaojie, Dongzhimen, Dongcheng District, Beijing 100700, China. Email: guojuanzy@163.com (JG), and huangluqi01@126.com (LH)

**Contents**

1. **Nucleotide sequence of *Sm2OGD25***…………………………….……………….4
2. **Amino acid sequence of Sm2OGD25**…...…………………………….………....4
3. **Supplementary Tables**……………………………………………..…….…...…..6

**Table S1.** Primers used in this study.………………………………………….…..6

**Table S2.** Chromatographic gradient elution condition of UPLC-QTOF-MS for functional characterization.………………………………………………...….......8

**Table S3.** ^1^H (600 MHz) and ^13^C NMR (150 MHz) data of hypargenin B (**1a**) and crossogumerin C (**1b**) in CDCl_3_ (*δ* in ppm and *J* in Hz).............................................9

**Table S4.** Experiments on the dependence of Sm2OGD25 on 2OG and Fe^2+^.........10

**Table S5.** The accession numbers of DOXC family proteins for phylogenetic analysis……………………………………………………………………..….…11

1. **Supplementary Figures** …………………………………………………….......15

**Figure S1.** Candidate substrate of Sm2OGD25.………………….………....…....15

**Figure S2.** ^1^H NMR spectrum of **1a** (600 MHz, CDCl_3_)……………….…......…..16

**Figure S3.** ^13^C NMR spectrum of **1a** (150 MHz, CDCl_3_)…………………..……..17

**Figure S4.** ^1^H NMR spectrum of **1b** (600 MHz, CDCl_3_)…………………….....…18

**Figure S5.** ^13^C NMR spectrum of **1b** (150 MHz, CDCl_3_)……....…...……............19

**Figure S6.** UPLC analysis of enzymatic reactions catalyzed by Sm2OGD25 as listed Table S4....………………………..………………………..………………20

**Figure S7.** Conserved domain prediction of Sm2OGD25…………………....…..21

**Figure S8.** Sequence Alignment of Sm2OGD25……………….………………..22

1. **Nucleotide sequence of *Sm2OGD25***

ATGGCTCCAGCTCCAATCTCAGGCATCAAAGTAGGCCACATAGATGATGTTCAAGAGCTAAGAAGAGGTGGGAAATCCTCACACATCCCTGCAAGATTCATCCGCGACACCACGGAGAGGCCGGCCCTAGACAAGGCCATCTTCTGCTCAGACACCATCCCTGTCATTGATCTCTCAAAACTCCACAAAGGAAGCAGTGATGAAATGCACAAGCTCATGAGCTCTTGTCAAGAATGGGGATTCTTTCAGGTTGTGAATCATGGGGTTGATGTGGAGTTGGTTGAGGGGATAGAGAGAGTGGCTATGGAGTTCTTCAAGATGCCTTTGGAGGAGAAGCAGAAGTATCCCATGAATCCTGGTACTGTACAAGGTTATGGTCAGGCCTTCATCTTCTCTGAAGATCAGAAATTGGATTGGTGCAACATGTTTGCACTTGGTGTTATACCAGACTACATTAGGAATCCAAAGCTCTGGCCATCTAAACCAGCAGATTTCAGTGAGACTGTTGACACATACTCAACACAAATAAGGTTACTGTGCAAGAATCTGCTGAAACACATAGCCACAACACTTGCACTAAAAGAGGATGTTTTTGAGGAGATGTTTGGAGTGGCTGTGCAAGCAGTGAGGATGAACTACTACCCGGCTTGCCCGAGGCCGGACCTCGTCTTAGGGCTAAGCCCGCACTCCGATGGTAGTGCCCTCACAGTTTTGCAACAGGCGAAGGGCAGCTCAGTTGGTCTCCAAATACTGAAAGATGGTAAATGGATATCAATTCAGCCCATCCCAAATGCTCTAGTCATCAACATTGGAGATACAATTGAGGTTTTGACTAATGGGAGATACAAGAGTGTGGAGCATAGAGCAGTGACTCACAAGGAGAAGGATAGGCTGTCTATAGTGACATTTTATGCTCCGAGCTACGATATCGAGCTCGGTCCATTGCACGAGTTTGTAGATGAGAATAATCCTTGCAAGTATAGGACATACAACCATGGAGAGTATAGCAAACACTATGTTACTAACAAGCTGCAGGGGAAGAAGGGGTTGGAATTTGCTAAGATTGTCAACTAA

1. **Amino acid sequence of Sm2OGD25**

MAPAPISGIKVGHIDDVQELRRGGKSSHIPARFIRDTTERPALDKAIFCSDTIPVIDLSKLHKGSSDEMHKLMSSCQEWGFFQVVNHGVDVELVEGIERVAMEFFKMPLEEKQKYPMNPGTVQGYGQAFIFSEDQKLDWCNMFALGVIPDYIRNPKLWPSKPADFSETVDTYSTQIRLLCKNLLKHIATTLALKEDVFEEMFGVAVQAVRMNYYPACPRPDLVLGLSPHSDGSALTVLQQAKGSSVGLQILKDGKWISIQPIPNALVINIGDTIEVLTNGRYKSVEHRAVTHKEKDRLSIVTFYAPSYDIELGPLHEFVDENNPCKYRTYNHGEYSKHYVTNKLQGKKGLEFAKIVN

1. **Supplementary Tables**

**Table S1.** Primers used in this study.

| **Primes** | **Sequences (5'-3')** |
| --- | --- |
| pET28a-Sm2OGD25-*Bam*HI-F | ggacagcaaatgggtcgcggatccATGGCTCCAGCTCCAATCTCAGGC |
| pET28a-Sm2OGD25-*Hin*dIII-R | ctcgagtgcggccgcaagcttTTAATTAGTTGACAACTTAGCAAATTCC |
| H229A-F | CGTCTTAGGGCTAAGCCCGgcCTCCGATGGTAG |
| H229A-R | gcCGGGCTTAGCCCTAAGACGAGGTCGGGCCTC |
| D231A-F | CTAAGCCCGCACTCCGcTGGTAGTGCC |
| D231A-R | gCGGAGTGCGGGCTTAGCCCTAAGACG |
| Y339A-F | GAGTATAGCAAACACgcTGTTACTAACAAG |
| Y339A-R | gcGTGTTTGCTATACTCTCCATGGTTG |
| F303A-F | ATAGGCTGTCTATAGTGACAgcTTACGCTCCGAG |
| F303A-R | gcTGTCACTATAGACAGCCTATCCTTCTCCTTGTG |
| V122A-F | TCCCATGAATCCTGGTACTGcACAAGGTTATGG |
| V122A-R | gCAGTACCAGGATTCATGGGATACTTCTGCTTC |
| V122L-F | ATCCCATGAATCCTGGTACTcTACAAGGTTATG |
| V122L-R | gAGTACCAGGATTCATGGGATACTTCTGCTTCTC |
| V122Y-F | TCCCATGAATCCTGGTACTtatCAAGGTTATGGTC |
| V122Y-R | ataAGTACCAGGATTCATGGGATACTTCTGCTTCTC |
| F129A-F | GGTTATGGTCAGGCCgcCATCTTCTCTG |
| F129A-R | gcGGCCTGACCATAACCTTGTACAGTAC |
| A144F-F | GATTGGTGCAACATGTTTttcCTTGGTGTTATAC |
| A144F-R | gaaAAACATGTTGCACCAATCCAATTTCTGATC |
| A144V-F | GATTGGTGCAACATGTTTGtACTTGGTGTTATAC |
| A144V-R | aCAAACATGTTGCACCAATCCAATTTCTGATC |
| A208L-F | GGTGTGGCTGTGCAActAGTGAGGATGAAC |
| A208L-R | agTTGCACAGCCACACCAAACATCTCC |
| A208S-F | TGTTTGGAGTGGCTGTGCAAtCAGTGAGGATGA |
| A208S-R | aTTGCACAGCCACTCCAAACATCTCCTCAAAAAC |
| A208T-F | TGTTTGGAGTGGCTGTGCAAaCAGTGAGGATGA |
| A208T-R | tTTGCACAGCCACTCCAAACATCTCCTCAAAAAC |
| L344A-F | CTATGTTACTAACAAGgcGCAGGGGAAG |
| L344A-R | gcCTTGTTAGTAACATAGTGTTTGCTATAC |

**Table S2.** Chromatographic gradient elution condition of UPLC-QTOF-MS for functional characterization.

| **Time (min)** | **Acetonitrile (Solvent A)** | **Water containing 0.1% formic acid (Solvent B)** |
| --- | --- | --- |
| 0 | 20% | 80% |
| 10.0 | 40% | 60% |
| 15.0 | 98% | 2% |
| 17.0 | 98% | 2% |
| 18.0 | 20% | 80% |
| 20.0 | 20% | 80% |

**Table S3.** ^1^H (600 MHz) and ^13^C NMR (150 MHz) data of hypargenin B (**1a**) and crossogumerin C (**1b**) in CDCl_3_ (*δ* in ppm and *J* in Hz).

| **1a** | | | | |  | **1b** | | | |
| --- | --- | --- | --- | --- | --- | --- | --- | --- | --- |
| **Position** | | | ***δ*_H_, multiplicity (*J*)** | ***δ*_C_** |  | **Position** | | ***δ*_H_, multiplicity (*J*)** | ***δ*_C_** |
| 1 | α | 1.52, m, overlapped | | 37.7 |  | 1 | α | 1.54, m | 37.8 |
|  | β | 2.25, d (12.8) | |  |  |  | β | 2.26, d (12.7) |  |
| 2 | α | 1.66, m | | 18.9 |  | 2 | α | 1.66, m | 18.9 |
|  | β | 1.77, m | |  |  |  | β | 1.76, m |  |
| 3 | α | 1.26, m | | 41.4 |  | 3 | α | 1.26, m | 41.4 |
|  | β | 1.52, m, overlapped | |  |  |  | β | 1.51, m |  |
| 4 |  |  | | 33.3 |  | 4 | |  | 33.3 |
| 5 |  | 1.83 dd (13.9, 3.9) | | 49.4 |  | 5 | | 1.85, m | 49.4 |
| 6 | α | 2.66, dd (18.1, 3.9) | | 36.0 |  | 6 | α | 2.68, dd (18.0, 4.1) | 36.1 |
|  | β | 2.58, dd (18.1, 13.5) | |  |  |  | β | 2.59, dd (18.0, 13.7) |  |
| 7 |  |  | | 198.5 |  | 7 | |  | 198.6 |
| 8 |  |  | | 123.5 |  | 8 | |  | 124.2 |
| 9 |  |  | | 158.3 |  | 9 | |  | 157.5 |
| 10 |  |  | | 38.0 |  | 10 | |  | 37.9 |
| 11 |  | 6.81, s | | 112.4 |  | 11 | | 6.86, s | 112.3 |
| 12 |  |  | | 161.1 |  | 12 | |  | 160.7 |
| 13 |  |  | | 129.1 |  | 13 | |  | 128.9 |
| 14 |  | 7.79, s | | 125.6 |  | 14 | | 7.83, s | 128.4 |
| 15 |  |  | | 76.4 |  | 15 | | 3.21, td (7.3, 2.9) | 37.3 |
| 16 |  | 1.68, s | | 30.5 |  | 16 | α | 4.01, dd (9.6, 3.3) | 69.4 |
|  |  |  |  |  |  |  | β | 3.78, dd (9.6, 3.0) |  |
| 17 |  | 1.70, s | | 30.6 |  | 17 | | 1.36, d (7.3) | 15.5 |
| 18 |  | 0.93, s | | 32.6 |  | 18 | | 0.93, s | 32.6 |
| 19 |  | 0.99, s | | 21.4 |  | 19 | | 0.99, s | 21.4 |
| 20 |  | 1.22, s | | 23.1 |  | 20 | | 1.22, s | 23.1 |

**Table S4.** Experiments on the dependence of Sm2OGD25 on 2OG and Fe^2+^.

| Reaction | 1  Normal | 2  Blank | 3  (-Sm2OGD25) | 4  (-2OG) | 5  (-L-ascorbic acid) | 6  (-Fe^2+^) | 7  (-Fe^2+^+ EDTA) | 8  (+EDTA) | 9  (-substrate) |
| --- | --- | --- | --- | --- | --- | --- | --- | --- | --- |
| sugiol (0.5mM) | √ | √ | √ | √ | √ | √ | √ | √ | DMSO |
| 2OG  (2 mM) | √ | √ | √ | — | √ | √ | √ | √ | √ |
| L-ascorbic acid (2 mM) | √ | √ | √ | √ | — | √ | √ | √ | √ |
| Fe^2+^  (0.1 mM) | √ | √ | √ | √ | √ | — | EDTA | √  EDTA | √ |
| Purified pET28a(+)- Sm2OGD25 | √ | pET28a(+) | — | √ | √ | √ | √ | √ | √ |
| reaction buffer (50 mM Tris-HCl, 300 mM NaCl, pH 7.5) up to 100 μL | | | | | | | | | |

**Table S5.** The accession numbers of DOXC family proteins for phylogenetic analysis.

| Name | Organism | Genbank accession number | Cluster^[45]^ |
| --- | --- | --- | --- |
| AtGA3ox1 | *Arabidopsis thaliana* | NP_173008 | DOXC3 |
| AtGA3ox2 | *Arabidopsis thaliana* | NP_178150 | DOXC3 |
| AtGA3ox3 | *Arabidopsis thaliana* | NP_193900 | DOXC3 |
| AtGA3ox4 | *Arabidopsis thaliana* | NP_178149 | DOXC3 |
| CmGA2,3ox | *Cucurbita maxima* | AAB64347 | DOXC3 |
| SmoGA3ox | *Selaginella moellendorffii* | ABX10776 | DOXC3 |
| AtGA20ox1 | *Arabidopsis thaliana* | NP_194272 | DOXC7 |
| AtGA20ox2 | *Arabidopsis thaliana* | NP_199994 | DOXC7 |
| AtGA20ox3 | *Arabidopsis thaliana* | NP_196337 | DOXC7 |
| AtGA20ox4 | *Arabidopsis thaliana* | NP_176294 | DOXC7 |
| AtGA20ox5 | *Arabidopsis thaliana* | NP_175075 | DOXC7 |
| CmGA20ox | *Cucurbita maxima* | AAB64345 | DOXC7 |
| SmoGA20ox | *Selaginella moellendorffii* | ABX10768 | DOXC7 |
| AtGA2ox1 | *Arabidopsis thaliana* | NP_177965 | DOXC12 |
| AtGA2ox2 | *Arabidopsis thaliana* | NP_174296 | DOXC12 |
| AtGA2ox3 | *Arabidopsis thaliana* | NP_181002 | DOXC12 |
| AtGA2ox4 | *Arabidopsis thaliana* | NP_175233 | DOXC12 |
| AtGA2ox6 | *Arabidopsis thaliana* | NP_171742 | DOXC12 |
| AtGA2ox7 | *Arabidopsis thaliana* | AEE32606 | DOXC13 |
| AtGA2ox8 | *Arabidopsis thaliana* | NP_193852.2 | DOXC13 |
| SoGA2ox3 | *Spinacia oleracea* | AAX14674 | DOXC13 |
| OsDAO | *Oryza sativa* | NP_001053075 | DOXC15 |
| AtAOP1 | *Arabidopsis thaliana Col* | NP_192216 | DOXC20 |
| AtAOP2 | *Arabidopsis thaliana Cvi* | AAL14646 | DOXC20 |
| AtAOP3 | *Arabidopsis thaliana Ler* | AAL14647 | DOXC20 |
| CmGA7ox | *Cucurbita maxima* | AAB64346 | DOXC22 |
| AcFNSI | *Aethusa cynapium* | ABG78791 | DOXC28 |
| AcF3H | *Aethusa cynapium* | ABG78792 | DOXC28 |
| AmF3H | *Ammi majus* | AAX21539 | DOXC28 |
| AgF3H | *Anethum graveolens* | AAX21540 | DOXC28 |
| AaFNSI | *Angelica archangelica* | ABG78793 | DOXC28 |
| AgFNSI | *Apium graveolens* | AAX21537 | DOXC28 |
| AtF3H | *Arabidopsis thaliana* | AEE78766 | DOXC28 |
| CmFNSI | *Conium maculatum* | AAX21538 | DOXC28 |
| CcFNSI | *Cuminum cyminum* | ABG78790 | DOXC28 |
| DcFNSI | *Daucus carota* | AAX21536 | DOXC28 |
| FaF3H | *Fragaria x ananassa* | AAU04791 | DOXC28 |
| GbF3H | *Ginkgo biloba* | AAU93347 | DOXC28 |
| GmF3H | *Glycine max* | AAT94365 | DOXC28 |
| MtF3H | *Medicago truncatula* | ACR15123 | DOXC28 |
| PcFNSI | *Petroselinum crispum* | AAP57393 | DOXC28 |
| PcF3H | *Petroselinum crispum* | AAP57394 | DOXC28 |
| PhF3H | *Petunia hybrida* | AAC49929 | DOXC28 |
| PaF3H | *Pimpinella anisum* | AAX21535 | DOXC28 |
| AtF6'H2 | *Arabidopsis thaliana* | NP_175925 | DOXC30 |
| AtF6'H1 | *Arabidopsis thaliana* | NP_187970 | DOXC30 |
| ClC2'H | *Citrus limetta* | AER36089 | DOXC30 |
| IbF6'H1 | *Ipomoea batatas* | BAL22344 | DOXC30 |
| IbC2'H | *Ipomoea batatas* | BAL22346 | DOXC30 |
| AtGSLOH | *Arabidopsis thaliana* | NP_180115 | DOXC31 |
| CrD4H | *Catharanthus roseus* | AAB97311 | DOXC31 |
| RsGSR1 | *Raphanus sativus* | XP_018482759 | DOXC31 |
| Sh2OGD | *Sinopodophyllum hexandrum* | ALG05126 | DOXC31 |
| ZmBX6 | *Zea mays* | NP_001105100 | DOXC31 |
| AtS3H | *Arabidopsis thaliana* | NP_192788 | DOXC38 |
| AaH6H | *Anisodus acutangulus* | ABM74185 | DOXC41 |
| AtaH6H | *Anisodus tanguticus* | AAQ75700 | DOXC41 |
| AbH6H | *Atropa baetica* | ABR15749 | DOXC41 |
| BcH6H | *Brugmansia candida* | ACB40931 | DOXC41 |
| DmH6H | *Datura metel* | AAQ04302 | DOXC41 |
| HvIDS2 | *Hordeum vulgare* | BAA03647 | DOXC41 |
| HvIDS3 | *Hordeum vulgare* | BAA75493 | DOXC41 |
| HnH6H | *Hyoscyamus niger* | AAA33387 | DOXC41 |
| MaH6H | *Mandragora autumnalis* | QJZ27966 | DOXC41 |
| MoH6H | *Mandragora officinarum* | QJZ27967 | DOXC41 |
| Sl16DOX | *Solanum lycopersicum* | BBD17782 | DOXC41 |
| St16DOX | *Solanum tuberosum* | BBD17781 | DOXC41 |
| AtJOX1 | *Arabidopsis thaliana* | NP_187728 | DOXC46 |
| AtJOX2 | *Arabidopsis thaliana* | Q9FFF6 | DOXC46 |
| AtJOX3 | *Arabidopsis thaliana* | Q9LY48 | DOXC46 |
| AtJOX4 | *Arabidopsis thaliana* | AEC09512 | DOXC46 |
| AtJRG1 | *Arabidopsis thaliana* | NP_191156 | DOXC46 |
| AcANS | *Allium cepa* | ABM66367 | DOXC47 |
| AtFLS1 | *Arabidopsis thaliana* | NP_196481 | DOXC47 |
| AtFLS3 | *Arabidopsis thaliana* | NP_201164 | DOXC47 |
| AtFLS5 | *Arabidopsis thaliana* | NP_001032131 | DOXC47 |
| AtLDOX | *Arabidopsis thaliana* | NP_194019 | DOXC47 |
| CitFLS | *Citrus unshiu* | BAA36554 | DOXC47 |
| FaFLS | *Fragaria x ananassa* | AAZ78661 | DOXC47 |
| FaANS | *Fragaria x ananassa* | AAU12368 | DOXC47 |
| GbFLS | *Ginkgo biloba* | ACY00393 | DOXC47 |
| GbANS | *Ginkgo biloba* | ACC66092 | DOXC47 |
| InANS | *Ipomoea nil* | BAB71811 | DOXC47 |
| McDAH | *Menispermum canadense* | QJD15033 | DOXC47 |
| NB17FLS | *Nierembergia sp.* | BAC10995 | DOXC47 |
| OsANS | *Oryza sativa* | CAA69252 | DOXC47 |
| PfANS | *Perilla frutescens* | BAA20143 | DOXC47 |
| PcFLS | *Petroselinum crispum* | AAP57395 | DOXC47 |
| PhFLS | *Petunia hybrida* | CAA80264 | DOXC47 |
| PaANS | *Phytolacca americana* | BAE54521 | DOXC47 |
| RhFLS | *Rosa hybrida* | BAC66468 | DOXC47 |
| SaDAH | *Sinomenium acutum* | QJD15032 | DOXC47 |
| SoANS | *Spinacia oleracea* | BAE54520 | DOXC47 |
| TfFLS | *Torenia fournieri* | BAC10995 | DOXC47 |
| ZmA2 | *Zea mays* | CAA39022 | DOXC47 |
| AtSRG1 | *Arabidopsis thaliana* | NP_173145 | DOXC52 |
| CjNCS | *Coptis japonica* | BAF45337 | DOXC52 |
| PsCOD | *Papaver somniferum* | ADD85331 | DOXC52 |
| PsDIOX4 | *Papaver somniferum* | AGL52586 | DOXC52 |
| PsDIOX5 | *Papaver somniferum* | XP_026450346 | DOXC52 |
| PsDIOX6 | *Papaver somniferum* | AGL52588 | DOXC52 |
| PsT6OD | *Papaver somniferum* | ADD85329 | DOXC52 |
| PsPODA | *Papaver somniferum* | XP_026429598 | DOXC52 |
| PtSRG1 | *Populus trichocarpa* | XP_002300453 | DOXC52 |
| RcSRG1 | *Ricinus communis* | XP_002519761 | DOXC52 |
| AtACO4 | *Arabidopsis thaliana* | NP_171994 | DOXC53 |
| AtACO3 | *Arabidopsis thaliana* | NP_172665 | DOXC53 |
| AtACO2 | *Arabidopsis thaliana* | NP_176428 | DOXC53 |
| AtACO5 | *Arabidopsis thaliana* | NP_565154 | DOXC53 |
| AtACO1 | *Arabidopsis thaliana* | NP_179549 | DOXC53 |
| MdACO1 | *Malus domestica Borkh.cv. Golden deliciou* | Q00985 | DOXC53 |
| SlACO1 | *Solanum lycopersicum* | NP_001234024.2 | DOXC53 |
| SmACO | *Salvia miltiorrhiza* | AFJ75398 | DOXC53 |
| At2OGD | *Arabidopsis thaliana* | NP_566685 | DOXC54 |
| ZmFLS/F3H | *Zea mays* | NM_001357949 | DOXC54 |

**Supplementary Figures**

**Figure S1.** Candidate substrate of Sm2OGD25.

**Figure S2.** ^1^H NMR spectrum of **1a** (600 MHz, CDCl_3_).

**Figure S3.** ^13^C NMR spectrum of **1a** (150 MHz, CDCl_3_).

**Figure S4.** ^1^H NMR spectrum of **1b** (600 MHz, CDCl_3_).

**Figure S5.** ^13^C NMR spectrum of **1b** (150 MHz, CDCl_3_).

**Figure S6.** UPLC analysis of enzymatic reactions catalyzed by Sm2OGD25 as listed Table S4.

**Figure S7.** Conserved domain prediction of Sm2OGD25. The conserved domain of amino acid sequence was performed by the InterProScan (http://www.ebi.ac.uk/interpro/search/sequence-search).

**Figure S8.** Sequence Alignment of Sm2OGD25.
